# Circular RNA circ-PVT1 contributes to paclitaxel resistance of gastric cancer cells through the regulation of ZEB1 expression by sponging miR-124-3p

**DOI:** 10.1042/BSR20193045

**Published:** 2019-12-24

**Authors:** Yan-yan Liu, Li-ying Zhang, Wen-zhen Du

**Affiliations:** 1 Department of General Surgery, Yantaishan Hospital, China; 2 Department of Pharmacy, Weihai Stomatological Hospital, China; 3 Department of Gastroenterology, Yantai YEDA Hospital, China

**Keywords:** apoptosis, circ-PVT1, invasion, miR-124-3p, paclitaxel, ZEB1

## Abstract

Gastric cancer (GC) is the fifth most commonly diagnosed malignancy. Paclitaxel (PTX) is an effective first-line chemotherapy drug in GC treatment, but the resistance of PTX attenuates the therapeutic effect. Circular RNA circ-PVT1 can exert the oncogenic effect in GC. But the function of circ-PVT1 involved in PTX resistance of GC is still unknown. In the present study, the expression levels of circ-PVT1, miR-124-3p and ZEB1 in PTX-resistant GC tissues and cells were detected by quantitative real-time polymerase chain reaction (RT-qPCR). PTX resistance in PTX-resistant cells was assessed by 3-(4,5-dimethyl-2-thiazolyl)-2,5-diphenyl-2-H-tetrazolium bromide (MTT) assay. The protein levels of Zinc finger E-box binding homeobox 1 (ZEB1), P-glycoprotein (P-gp) and glutathione S-transferase (GST-π) were detected by Western blot assay. Cell apoptosis and invasion were measured in PTX-resistant cells by flow cytometry and transwell invasion assays, severally. The interaction between miR-124-3p and circ-PVT1 or ZEB1 was predicted by starBase software, and then verified by the dual-luciferase reporter assay. The role of circ-PVT1 in PTX resistance of GC *in vivo* was measured by xenograft tumor model. Our results showed that circ-PVT1 expression was up-regulated in PTX-resistant GC tissues and cells. Circ-PVT1 down-regulation enhanced PTX sensitivity in PTX-resistant GC cells by negatively regulating miR-124-3p. ZEB1 served as a direct target of miR-124-3p. Circ-PVT1 enhanced ZEB1 expression by sponging miR-124-3p. Circ-PVT1 knockdown increased PTX sensitivity of GC *in vivo.* Taken together, our studies disclosed that circ-PVT1 facilitated PTX resistance by up-regulating ZEB1 mediated via miR-124-3p, suggesting an underlying therapeutic strategy for GC.

## Introduction

Gastric cancer (GC) is the third leading cause of cancer-related death and the fifth most commonly diagnosed malignancy, accounting for 1 in 12 deaths cases in the world in 2018 [1]. Although the development of surgical techniques and treatment has substantially improved, the prognosis of GC patients in China is still unfavorable [2]. Paclitaxel (PTX, also known as taxol) is identified as an effective first-line chemotherapy drug in GC treatment, but the therapeutic effect of PTX gradually become worse, on account of the chemoresistance caused by diverse factors [3,4]. Hence, it is imperative to identify the molecular mechanisms of drug resistance in GC to develop the novel molecular target for improving PTX sensitivity.

Circular RNAs (termed circRNAs), a class of special non-coding transcripts, have been reported as essential regulators in various biological processes of eukaryotes [5,6]. Moreover, some reports displayed that circRNAs were closely related to chemoresistance development in numerous tumors [7,8]. A circular RNA derived from one exon of the PVT1 gene (known as circ-PVT1) has been reported as a carcinogenic factor in GC, suggesting the oncogenic effect of circ-PVT1 in various cancers. For example, circ-PVT1 performed as a competing endogenous RNA (ceRNA) in oral squamous cell carcinoma (OSCC) to facilitate cell proliferation by regulating miR-125b/STAT3 axis [9]. Consistently, abundance of circ-PVT1 contributed to the malignancy of non-small cell lung cancer by boosting proliferation and invasion through activating E2F2 signaling [10]. Similarly, relevant research suggested the high expression of circ-PVT1 was a proliferative factor and prognostic marker in GC [11]. Furthermore, a recent publication reported that circ-PVT1 could promote the resistance of doxorubicin and cisplatin by regulating ABCB1 in osteosarcoma cells [12]. However, the biological function of circ-PVT1 involved in PTX resistance of GC is largely unknown.

MicroRNAs (miRNAs), with length of 19–25 nucleotides, have been reported to negatively regulate gene expression by binding to the 3′-untranslated region (3′UTR) of target mRNAs [13]. Mounting evidence indicated that miRNAs participated in regulating the formation and development of GC [14–16]. MiR-124-3p, a form of mature miR-124, is identified as a tumor suppressor by repressing proliferation, migration, invasion and promoting apoptosis in GC [17]. Also, a recent study has proved that miR-124-3p was associated with chemo-sensitivity of chronic myeloid leukemia [18]. Nevertheless, the involvement of miR-124-3p in the PTX sensitivity remains unclear in GC.

Zinc finger E-box binding homeobox 1 (ZEB1) has been demonstrated as a crucial transcriptional inhibitor of E-cadherin that expedites epithelial–mesenchymal transition (EMT), thereby accelerating the migration and invasiveness in various tumors, including GC [19–21]. Synchronously, ZEB1 was found to induce chemoresistance to PTX in ovarian cancer [22]. To our knowledge, the correlation between ZEB1 expression and PTX resistance in GC has not been reported.

Herein, our results showed that circ-PVT1 was highly expressed and the knockdown of circ-PVT1 improved PTX sensitivity in PTX-resistant GC cells. Importantly, circ-PVT1 could bind to miR-124-3p as a miRNA sponge to up-regulate ZEB1 expression, thereby expediting paclitaxel resistance of gastric cancer cells. Taken together, our findings illuminated the involvement of circ-PVT1/miR-124-3p/ZEB1 axis in the PTX resistance of GC.

## Materials and methods

### Clinical samples and cell culture

Samples of the 30 paclitaxel (PTX)-sensitive patients and 30 PTX-resistant patients were obtained from GC patients who underwent a surgical operation at Yantaishan Hospital. The present study was authorized by the Ethical Committee of Yantaishan Hospital. All participants signed the written informed consents. All the experiments were performed at Yantaishan Hospital and were in accordance with the Declaration of Helsinki Principles.

Human GC tumor cell lines (MKN-45, HGC-27, MGC-803, and AGS) were acquired from Otwo Biotech Co., Ltd. (Shenzhen, China). Normal human gastric epithelial cell line (GES-1) was obtained from Innovation Biotechnology Co., Ltd. (Shanghai China). All GC tumor cells were maintained in 89% Roswell Park Memorial Institute 1640 (RPMI-1640, Hyclone, Beijing, China) medium containing 10% fetal bovine serum (FBS; Hyclone) and 1% antibiotic (100 U/ml penicillin and 100 μg/ml streptomycin, Invitrogen, Carlsbad, CA, U.S.A.). GES-1 cells were cultured in Dulbecco’s modified Eagle’s medium (DMEM) supplemented with 10% FBS (Hyclone) and antibiotics (Invitrogen). All cells were incubated in a 37°C atmosphere with 5% CO_2_. Besides, 5 nM PTX was added into the culture medium of MGC-803/PTX and AGS/PTX cells for keeping the PTX-resistant phenotype.

### Cell transfection

circ-PVT1 or ZEB1 overexpression vector was generated by introducing circ-PVT1 or ZEB1 cDNA sequence into pcDNA3.1 empty vector (pcDNA, Addgene, Inc., Cambridge, MA, U.S.A.), termed circ-PVT1-pcDNA3.1 (circ-PVT1), ZEB1-pcDNA3.1 (ZEB1). Short hairpin RNA (shRNA) against circ-PVT1 (sh-circ-PVT1) shRNA against ZEB1 (sh-ZEB1) and their negative control (sh-NC) were purchased from GeneCopoeia (Rockville, MD, U.S.A.). MiR-124-3p mimic (miR-124-3p), miR-124-3p inhibitor (anti-miR-124-3p), and their corresponding negative control (miR-NC, anti-miR-NC) were purchased from GenePharma (Suzhou, China). Transfection of all plasmids and oligonucleotides was carried out by the Lipofectamine 3000 reagents (Invitrogen).

### RNA extraction and quantitative real-time PCR (RT-qPCR)

Extraction of RNA from tissues and cells was enforced in line with the user’s guidebook of TRIzol reagent (Gibco, Carlsbad, CA, U.S.A.). Total RNA was purified with an RNeasy Mini Elute Cleanup Kit (Qiagen, Waltham, MA, U.S.A.). The first-strand complementary DNA (cDNA) of circ-PVT1, ZEB1, P-glycoprotein (P-gp) and glutathione S-transferase (GST-π) was synthesised by PrimeScript Reverse Transcription Kits (Takara, Shiga, Japan). The cDNA of miR-124-3p was synthesised by MicroRNA Reverse Transcription Kit (Applied Biosystems, Foster City, CA, U.S.A.). The relative expression of target genes was tested by the SYBR Premix DimerEraser Kit (Takara) On a CFX96 Sequence Detection System (BioRad, Berkeley, CA, U.S.A.). The levels of target gene were calculated using 2^–△△*C*^_t_ method, with glyceraldehyde-3-phosphate dehydrogenase (GAPDH) and U6 small nuclear RNA (snRNA) as endogenous controls, respectively. There sequences were as followed:

Circ-PVT1 (Forward: 5′-GGTTCCACCAGCGTTATTC-3′, Reverse: 5′-CAACTTCCTTTGGGTCTCC-3′);
MiR-124-3p (Forward: 5′-ACACTCCAGCTGGGTAAGGCACGCGGTG -3′, Reverse: 5′-TGGTGTCGTGGAGTCG-3′);
ZEB1 (Forward: 5′-CAGCTTGATACCTGTGAATGGG -3′, Reverse: 5′-TATCTGTGGTGTGGGACT-3′);
P-gp (Forward: 5′-GAATGTTCAGTGGCTCCGAG -3′, Reverse: 5′-ACAATCTCTTCCTGTGACACC-3′);
GST-π (Forward: 5′-ATACCATCCTGCGTCACCTG -3′, Reverse: 5′-TCCTTGCCCGCCTCATAGTT-3′);
U6 (Forward: 5′-CTCGCTTCGGCAGCACA-3′, Reverse: 5′-AACGCTTCACGAATTTGCGT-3′);
GAPDH (Forward: 5′-TCCACCACCCTGTTGCTGTA-3′, Reverse: 5′-ACCACAGTCCATGCCATCAC-3′).

### Drug resistance assay

3-(4,5-dimethyl-2-thiazolyl)-2,5-diphenyl-2-H-tetrazolium bromide (MTT) assay was used to test the PTX resistance referring to the operation manual. First, incubated MGC-803/PTX and AGS/PTX cells were exposed with different doses PTX for 48 h. Following adding with 20 μl MTT (5 mg/ml, Gibco) for 4 h, 150 μl dimethyl sulfoxide (DMSO, Xiya Chemical Industry Co., Ltd, Shandong, China) was added to dissolve the formed formazan crystals. The absorbance of the cells at 490 nm was assessed with a microplate reader. The 50% inhibition of growth (IC50) was displayed by using the relative survival curve.

### Western blot assay

The assay was implemented in line with the previous description [23]. Generally, the separated proteins were and transferred onto polyvinylidene fluoride (PVDF) membranes (Millipore, Bedford, MA, U.S.A.). Then, the membranes were blocked with 5% non-fat milk. Afterwards, the membranes were incubated with primary antibodies, including ZEB1 (1:1000, ab181451; Abcam, Cambridge, MA, U.S.A.), P-gp (1:1000, ab3364; Abcam) and GST-π (1:800, ab47709; Abcam) and GAPDH (1:8000, ab8245, Abcam), followed by probed with corresponding secondary antibody (Abcam) to combine these primary antibodies. At last, proteins blots were assessed with an enhanced chemiluminescence detection kit (Millipore).

### Cell apoptosis assay

Transfected MGC-803/PTX and AGS/PTX cells were harvested and washed with pre-cooled phosphate-buffered saline (PBS, Invitrogen). After resuspending in 100 μl binding buffer, MGC-803/PTX and AGS/PTX cells were incubated with 5 μl (Annexin V-fluorescein isothiocyanate) Annexin V-FITC and Propidium Iodide (PI) (KeyGen Biotech, Nanjing, China) based on the operation manual. The ratio of apoptotic GC cells was subjected to a FACSCalibur (BD Bioscience, San Jose, CA, U.S.A.).

### Cell invasion assay

Cell invasion ability was tested by transwell invasion assay referring to the user’s guidebook. Transfected MGC-803/PTX and AGS/PTX cells were collected and resuspended in serum-free RPMI-1640 medium (Gibco). And then, these cells were introduced into the upper chamber coated with matrigel (BD Bioscience). The lower chamber was filled up with RPMI-1640 medium (Gibco) with 10% FBS (Gibco) as a chemoattractant. At 24 h post-incubation, non-invasive cells inside the upper chamber were scraped off with a cotton swab. The cells invaded to the lower chamber were fixed in methanol for 15 min and stained with Crystal Violet for 20 min. At last, cells were photographed with an inverted microscope.

The fragment of circ-PVT1 or ZEB1 3′UTR harboring wild-type or mutant-type predicted miR-124-3p binding site was amplified and inserted into the firefly luciferase reporter psiCHECK-2 vector (Promega, Madison, WI, U.S.A.), termed WT-circ-PVT1, MUT-circ-PVT1, ZEB1 3′UTR-WT, and ZEB1 3′UTR-MUT reporter vectors. Subsequently, MGC-803/PTX and AGS/PTX cells were co-transfected with the constructed reporter and miR-NC or miR-124-3p by the Lipofectamine 3000 reagents (Invitrogen). Luciferase activities at 48 h post-transfection were assessed in cell lysates by using a dual-luciferase reporter assay system (Promega).

### Tumor xenograft assay

For the stable expression of sh-circ-PVT1, the shRNA sequences of circ-PVT1 were inserted into lentivirus vector (Genechem, Shanghai, China), generating sh-circ-PVT1 lentivirus vector (lentivirus empty vector acted as sh-control). The animal experiment was carried out referring to the protocol approved by the Institutional Committee for Animal Research. Four-week-old male BALB/C nude mice (*n* = 6 per group) were collected from Hubei Research Center of Laboratory Animal (Wuhan, China). MGC-803/PTX cells were stably transfected with sh-circ-PVT1 or sh-control. Whereafter, the left flank of the nude mice was subcutaneously injected 4 × 10^6^ transduced cells. At 7 days after injection, sh-control + PBS and sh-circ-PVT1 + PBS groups were intraperitoneally administrated PBS, sh-control + PTX and sh-circ-PVT1 + PTX groups were intraperitoneally administrated 3 mg/kg PTX (Bristol-Myers Squibb, Shanghai, China) every 4 days. Tumor volume was examined every 4 days after the first injection. Twenty-seven days later, tumors were excised and weighed. Also, the circ-PVT1 expression level in xenograft tumors was detected by RT-qPCR assay.

### Statistical analysis

SPSS 17.0 software (IBM, Chicago, IL, U.S.A.) and Graph pad Prism 5.0 were employed for all statistical analysis. The difference of data between two groups or among multiple groups was analyzed with Student’s *t*-test or one-way analysis of variance (ANOVA). Data were showed as the mean ± standard deviation (SD) from at three experiments. The results were considered statistically significant at *P* value < 0.05.

## Results

### Circ-PVT1 was highly expressed in PTX-resistant GC tissues and cells

**Figure 1:**
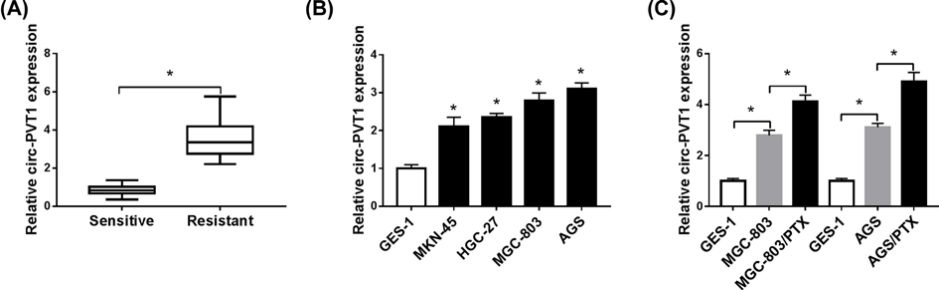
Circ-PVT1 was highly expressed in PTX-resistant GC tissues and cells (**A**) Expression of circPVT1 in 30 pairs of PTX-resistant GC tissues and adjacent PTX-sensitive GC tissues. (**B**) RT-qPCR assay was employed to detect the expression of circPVT1 in GC cell lines (MKN-45, HGC-27, MGC-803, and AGS) and normal human gastric epithelial cell line (GES-1). (**C**) Expression level of circPVT1 in PTX-resistant GC cells was measured by RT-qPCR assay; *P < 0.05.

Firstly, we investigated the expression level of circ-PVT1 in PTX-resistant GC tissues (*n* = 30) and adjacent PTX-sensitive GC tissues (*n* = 30) obtained from patients with GC. As displayed in Figure 1A, compared with PTX-sensitive tissues, circ-PVT1 expression was apparently increased in PTX-resistant GC tissues. Then, we also confirmed that circ-PVT1 was highly expressed in GC cell lines (MKN-45, HGC-27, MGC-803, and AGS) relative to normal human gastric epithelial cell line GES-1, particularly in MGC-803 and AGS cells (Figure 1B). Therefore, we selected H460 and A549 cells for the subsequent analyses. Furthermore, circ-PVT1 level in PTX-resistant GC cells (MGC-803/PTX and AGS/PTX) was further explored. Similarly, higher expression of circ-PVT1 was observed in PTX-resistant GC cells in contrast with their parental cells MGC-803 and AGS (Figure 1C). In a word, these results suggested that circ-PVT1 might be associated with PTX resistance in GC cells.

### Circ-PVT1 knockdown enhanced PTX sensitivity in PTX-resistant GC cells

**Figure 2:**
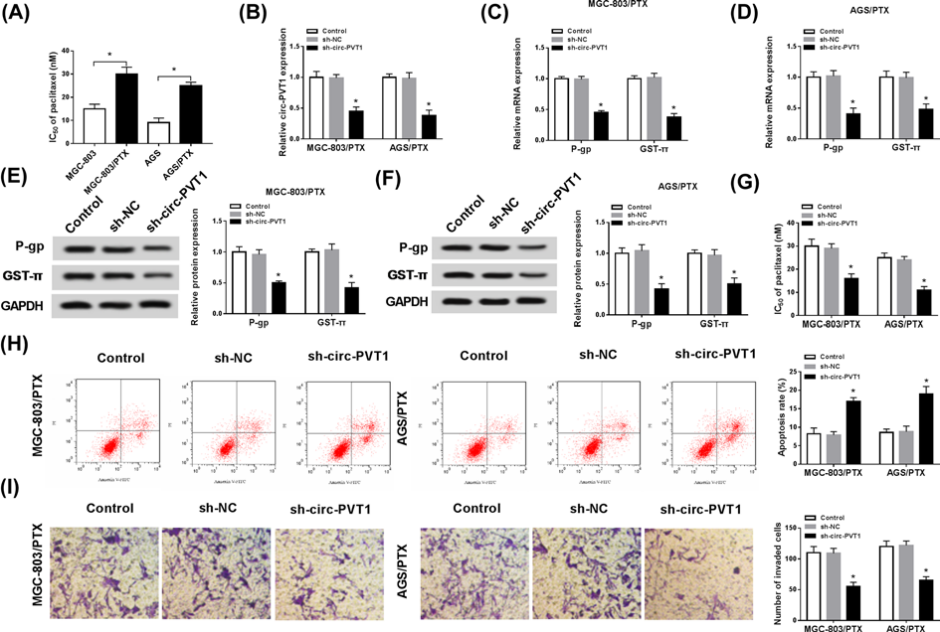
Circ-PVT1 knockdown enhanced PTX sensitivity in PTX-resistant gastric cancer cells (**A**) IC_50_ value of PTX in MGC-803, MGC-803/PTX, AGS, and AGS/PTX cells. (**B**) Transfection efficiency of sh-circPVT1 in MGC-803/PTX and AGS/PTX cells. (**C** and **D**) Expression levels of P-gp and GST-π were detected by RT-qPCR in MGC-803/PTX and AGS/PTX cells. (**E** and **F**) P-gp and GST-π expression levels were assessed by Western blot in MGC-803/PTX and AGS/PTX cells transfected with Control, sh-NC and sh-circPVT1. (**G**) IC_50_ value of PTX in transfected MGC-803/PTX and AGS/PTX cells. (**H**) Apoptosis rates were detected by flow cytometry assay in transfected MGC-803/PTX and AGS/PTX cells. (**I**) Invasion capability was assessed by transwell invasion assay in transfected MGC-803/PTX and AGS/PTX cells; *P < 0.05.

Next, to identify the effect of circ-PVT1 on PTX-resistant GC cells, IC_50_ value of PTX was first measured by MTT assay. As illustrated in Figure 2A, IC_50_ value of PTX was evidently elevated in MGC-803/PTX and AGS/PTX cells in comparison with MGC-803 and AGS cells, suggesting the production of PTX resistance in MGC-803/PTX and AGS/PTX cells. Then, to identify the effect of circPVT1 on PTX-resistant GC cells, the knockdown short hairpin RNA (shRNA) of circ-PVT1 was synthesized. The transfection efficiency was detected and presented in Figure 2B. Moreover, some studies have indicated that certain proteins were related to multidrug resistance (MDR), such as P-gp and GST-π [24,25]. Thus, we used the knockdown system to further explore the impact of circPVT1 on the levels of P-gp and GST-π. RT-qPCR analysis disclosed that mRNA levels of P-gp and GST-π were obviously repressed after down-regulation of circ-PVT1 in MGC-803/PTX and AGS/PTX cells (Figure 2C,D). Similarly, deletion of circ-PVT1 obviously also suppressed the protein levels of P-gp and GST-π in MGC-803/PTX and AGS/PTX cells (Figure 2E,F). Besides, IC_50_ determination displayed that knockdown of circ-PVT1 resulted in an evident decrease in PTX resistance in MGC-803/PTX and AGS/PTX cells (Figure 2G). Subsequently, to further probe the influence of circPVT1 on the progression of PTX-resistant GC cells, cell apoptosis and invasion were assessed. The results of flow cytometry pointed out that silencing of circ-PVT1 promoted PTX-triggered apoptosis in MGC-803/PTX and AGS/PTX cells (Figure 2H). Moreover, the promotion of invasion capability caused by PTX was impeded after introduction with sh-circ-PVT1 in MGC-803/PTX and AGS/PTX cells (Figure 2I). These data suggested that circ-PVT1 deficiency sensitized MGC-803/PTX and AGS/PTX cells to PTX.

### Circ-PVT1 knockdown improved PTX sensitivity in PTX-resistant GC cells by negatively regulating miR-124-3p

**Figure 3:**
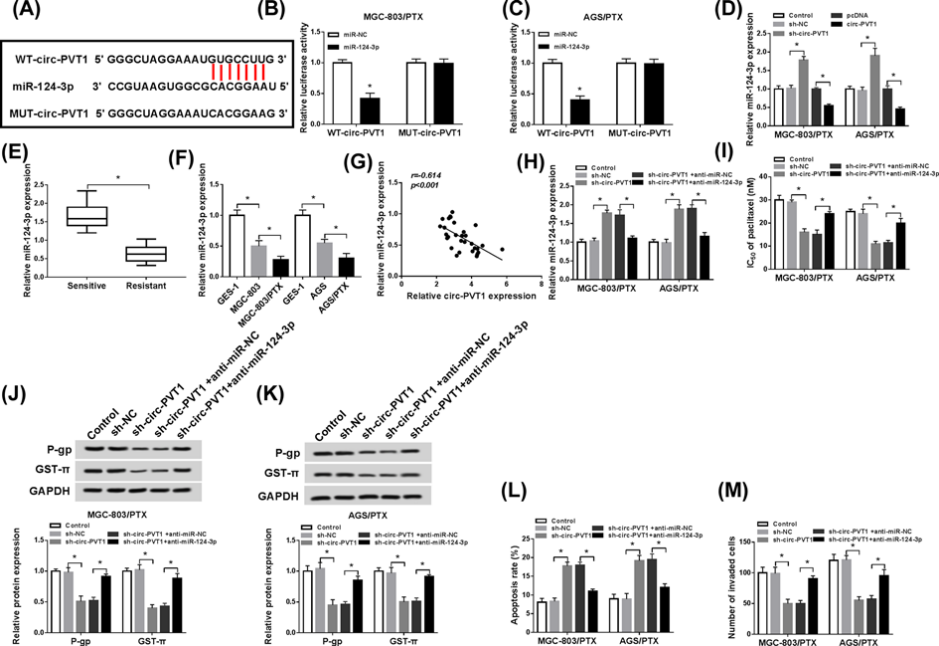
Circ-PVT1 knockdown improved PTX sensitivity in PTX-resistant GC cells by negatively regulating miR-124-3p (**A**) Predicted binding sites between circ-PVT1 and miR-124-3p, and mutant sites in MUT-circ-PVT1. (**B** and **C**) The effects of miR-124-3p overexpression on luciferase activity of WT-circ-PVT1, and MUT-circ-PVT1 reporters were measured in MGC-803/PTX and AGS/PTX cells. (**D**) MiR-124-3p expression was detected by RT-qPCR assay in MGC-803/PTX and AGS/PTX cells transfected with Control, sh-NC, sh-circ-PVT1, pcDNA, and circ-PVT1. (**E**) Expression level of miR-124-3p in 30 pairs of PTX-resistant GC tissues and adjacent PTX-sensitive GC tissues was measured by RT-qPCR assay. (**F**) MiR-124-3p expression was detected by RT-qPCR assay in GES-1, MGC-803, AGS, MGC-803/PTX, and AGS/PTX cells. (**G**) The correlation analysis between circ-PVT1 and miR-124-3p expression was analyzed using the Pearson’s correlation algorithm. (**H**) MiR-124-3p level was assessed by RT-qPCR assay in MGC-803/PTX and AGS/PTX cells transfected with Control, sh-NC, sh-circ-PVT1, sh-circ-PVT1 + anti-miR-NC, sh-circ-PVT1 + anti-miR-124-3p. (**I**) IC_50_ values of PTX were calculated by MTT assay in transfected MGC-803/PTX and AGS/PTX cells. (**J** and**K**) Western blot analysis of P-gp and GST-π expression levels in transfected MGC-803/PTX and AGS/PTX cells. (**L**) Flow cytometry analysis of apoptotic rate in transfected MGC-803/PTX and AGS/PTX cells. (**M**) Transwell analysis of invasion capability in transfected MGC-803/PTX and AGS/PTX cells; *P < 0.05.

It has been widely accepted that circ-RNA can exert its function by interacting with miRNA [26,27]. Through web-based tool starBase v2.0, miR-124-3p was found to have a motif with complementary sequence with circ-PVT1 (Figure 3A). To confirm the direct interaction between circ-PVT1 and miR-124-3p, the dual luciferase reporter assay was carried out. As presented in Figure 3B,C, miR-124-3p evidently decreased the luciferase activity of WT-circ-PVT1 reporter plasmid, but it had little effect on the luciferase activity of MUT-circ-PVT1 reporter plasmid in MGC-803/PTX and AGS/PTX cells. Meantime, RT-qPCR analysis showed that miR-124-3p expression was obviously up-regulated in sh-circ-PVT1-transfected MGC-803/PTX and AGS/PTX cells, and was notably down-regulated in pcDNA-circ-PVT1- transfected MGC-803/PTX and AGS/PTX cells (Figure 3D), suggesting that circ-PVT1 interacted with miR-124-3p to repress its expression. Not surprisingly, miR-124-3p was expressed at low level in PTX-resistant patients in contrast with that in PTX-sensitive patients (Figure 3E). Also, lower expression of miR-124-3p was viewed in PTX-resistant GC cells versus their matched MGC-803 and AGS cells (Figure 3F). That was to say, miR-124-3p was involved in PTX resistance in GC. Additionally, the negative correlation between miR-124-3p and circ-PVT1 also was verified in PTX-resistant GC tissues (Figure 3G). Then, to probe the mechanism of circ-PVT1 in PTX sensitivity, MGC-803/PTX and AGS/PTX cells were transfected with Control, sh-NC, sh-circ-PVT1, sh-circ-PVT1 + anti-miR-NC, sh-circ-PVT1 + anti-miR-124-3p. As illustrated in Figure 3H, knockdown of circ-PVT1 elevated miR-124-3p expression level in MGC-803/PTX and AGS/PTX cells, which was remarkably weakened after co-transfection with sh-circ-PVT1. The results of IC_50_ determination pointed out that miR-124-3p inhibition partly overturned the negatively effect of circ-PVT1 depletion on PTX resistance in MGC-803/PTX and AGS/PTX cells (Figure 3I). Similar to the IC_50_ determination results, the reintroduction of anti-miR-124-3p notably reversed sh-circ-PVT1-triggered reduction in expression levels of P-gp and GST-π in MGC-803/PTX and AGS/PTX cells (Figure 3J,K). Simultaneously, flow cytometry analysis disclosed that deficiency of circ-PVT1 expedited PTX-induced apoptosis, while silence of miR-124-3p effectively attenuated the promoting effect of sh-circ-PVT1 on PTX-induced apoptosis (Figure 3L). Transwell assay suggested that knockdown of circ-PVT1 partially relieved the PTX-caused positive effect on cell invasion capability and subsequent miR-124-3p down-regulation recovered cell invasion capability (Figure 3M). In summary, deletion of miR-124-3p partly abolished the promotion effect of circ-PVT1 down-regulation on PTX sensitivity in PTX-resistant GC cells.

### ZEB1 was a direct target of miR-124-3p

**Figure 4:**
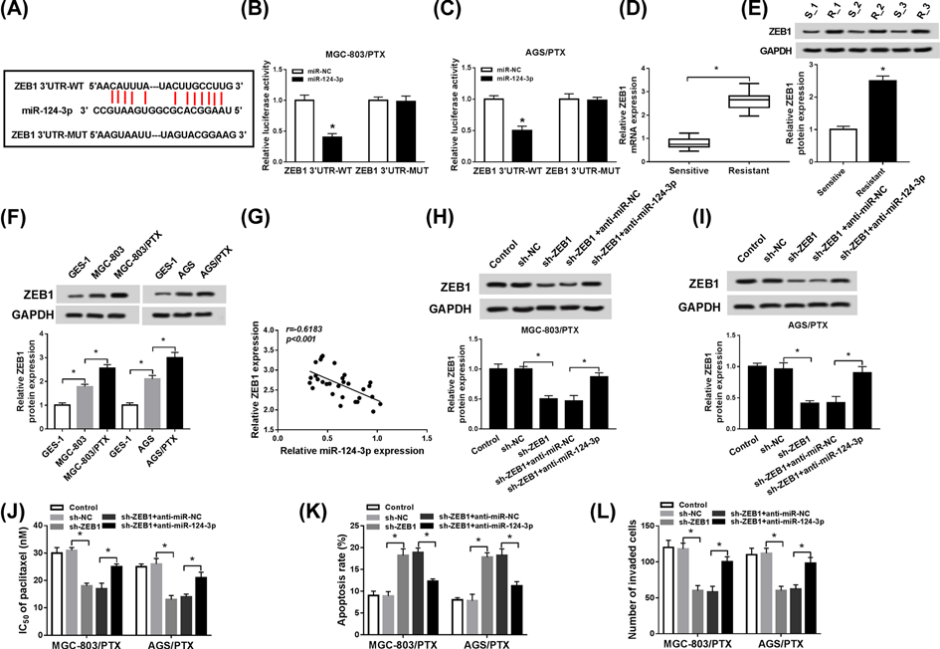
ZEB1 was a direct target of miR-124-3p in PTX-resistant GC cells (**A**) Putative binding regions between ZEB1 and miR-124-3p. (**B** and **C**) The interactions between ZEB1 and miR-124-3p were verified by luciferase reporter assay in MGC-803/PTX and AGS/PTX cells. (**D**) Expression of ZEB1 in 30 pairs of PTX-resistant GC tissues and adjacent PTX-sensitive GC tissues. (**E**) ZEB1 protein level was measured in PTX-resistant GC tissues and adjacent PTX-sensitive GC tissues (**F**) Western blot analysis of ZEB1 protein level in GES-1, MGC-803, MGC-803/PTX, AGS, and AGS/PTX cells. (**G**) Pearson’s correlation analysis was used to assess the expression association between ZEB1 and miR-124-3p in PTX-resistant GC tissues. (**H** and **I**) ZEB1 expression level was determined by Western blot assay in MGC-803/PTX and AGS/PTX cells transfected with Control, sh-NC, sh-ZEB1, sh-ZEB1 + anti-miR-NC and sh-ZEB1 + anti-miR-124-3p. (**J**) IC_50_ value of PTX in transfected MGC-803/PTX and AGS/PTX cells. (**K**) Apoptosis rates in transfected MGC-803/PTX and AGS/PTX cells were measured by flow cytometry assay. (**L**) Invasion capability in transfected MGC-803/PTX and AGS/PTX cells was detected by transwell invasion assay; *P < 0.05.

As widely believed, functional mechanism of miRNA depends on its specific binding to the 3′-UTR of the downstream genes. Therefore, starBase v2.0 prediction software was used to search the potential downstream target gene of miR-124-3p. As seen in Figure 4A, 3′-UTR of ZEB1 possessed some complementary sequences to miR-124-3p. Meantime, we viewed that the luciferase activity was distinctly reduced in MGC-803/PTX and AGS/PTX cells co-transfected with ZEB1-WT and miR-124-3p, but he luciferase activity changed little in cells co-transfected with ZEB1-MUT and miR-124-3p (Figure 4B,C), demonstrating the possible interaction between ZEB1 and miR-124-3p. Previous reporters showed that ZEB1 played a carcinogenic role in GC development [19], which also proved by our paper and displayed in Figure 4D,E. Both mRNA level and protein level of ZEB1 were increased in PTX-sensitive GC tissues. Importantly, we observed that ZEB1 was expressed at high level in MGC-803/PTX and AGS/PTX cells compared with that in MGC-803 and AGS cells (Figure 4F), meaning the involvement of ZEB1 in PTX resistance in GC. Then, we further explored whether miR-124-3p could exert its function in PTX sensitivity by regulating ZEB1 in PTX-resistant GC cells. Firstly, we testified that miR-124-3p expression level was negatively associated with ZEB1 expression level in PTX-resistant GC tissues (Figure 4G). Subsequently, we found that miR-124-3p knockdown of miR-124-3p apparently enhanced the expression of ZEB1, which was mitigated after co-introduction with anti-miR-124-3p in MGC-803/PTX and AGS/PTX cells (Figure 4H,I). Also, IC_50_ value of PTX determination indicated that miR-124-3p down-regulation partly relieved the inhibitory effect of ZEB1 deficiency on PTX resistance in MGC-803/PTX and AGS/PTX cells (Figure 4J). Not surprisingly, transfection of sh-ZEB1 contributed to PTX-caused apoptosis in MGC-803/PTX and AGS/PTX cells, while silence of miR-124-3p obviously overturned the effect (Figure 4K). Additionally, low expression of ZEB1 led to an overt suppression of PTX-triggered invasion capability in MGC-803/PTX and AGS/PTX cells and subsequently miR-124-3p down-regulation recuperated cell invasion capability (Figure 4L). Collectively, these data revealed that miR-124-3p contributed to PTX sensitivity in PTX-resistant GC cells by targeting ZEB1.

### Circ-PVT1 elevated ZEB1 expression by sponging miR-124-3p

**Figure 5:**
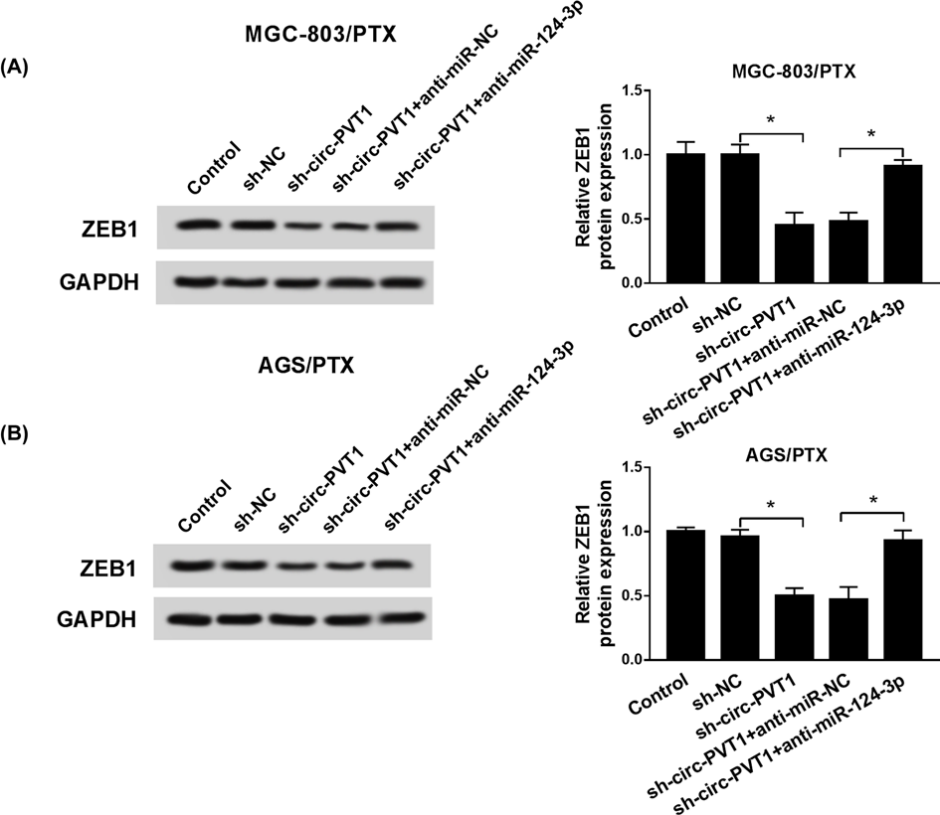
Circ-PVT1 elevated ZEB1 expression by sponging miR-124-3p in PTX-resistant GC cells (**A** and **B**) ZEB1 expression level was monitored by Western blot in MGC-803/PTX and AGS/PTX cells transfected with Control, pcDNA, circ-PVT1, circ-PVT1 + miR-NC, and circ-PVT1 + miR-124-3p; *P < 0.05.

Additionally, we implemented rescue experiment to validate whether circ-PVT1 could influence the expression of ZEB1 by miR-124-3p in PTX resistance of GC cells. Western blot analysis verified that the exogenous expression of circ-PVT1 resulted in an enhancement of ZEB1 protein expression, and recovery of miR-124-3p expression effectively abated the effect of circ-PVT1 on the expression of ZEB1 in MGC-803/PTX and AGS/PTX cells (Figure 5A,B). Together, these data manifested that circ-PVT1 served as a molecular sponge to sequester miR-124-3p from ZEB1 in PTX-resistant GC cells.

### Circ-PVT1 knockdown improved PTX sensitivity of GC *in vivo*

**Figure 6:**
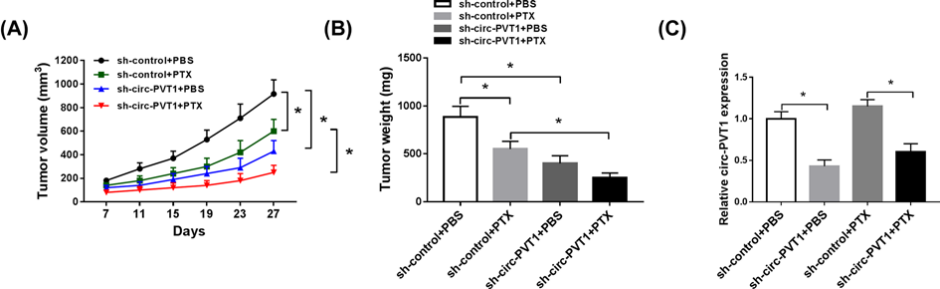
Circ-PVT1 knockdown improved PTX sensitivity of GC *in vivo* (**A**) Tumour volume was measure at the indicated time points (day 7, 11, 15, 19, 23, and 27). (**B**) At 27 days post cell implantation, tumours were excised and weighted. (**C**) Circ-PVT1 expression level was detected by RT-qPCR assay in xenograft tumors; *P < 0.05.

Finally, a mice xenograft model of GC was established to explore the effect of circ-PVT1 on PTX resistance *in vivo*. According to the results exhibited in Figure 6A,B, tumor size and weight were declined in presence of PTX treatment or circ-PVT1 knockdown, implicating that PTX treatment or circ-PVT1 silencing repressed tumor growth of CG *in vivo*. Interestingly, we viewed that circ-PVT1 down-regulation reinforced PTX-induced anti-tumor effect *in vivo*. Furthermore, RT-qPCR results confirmed that circ-PVT1 expression was strikingly dropped in sh-circ-PVT1 group when compared with sh-control group (Figure 6C). In other words, circ-PVT1 knockdown induced PTX sensitivity of GC *in vivo*.

## Discussion

With rapid development in high-throughput sequencing, many circRNAs have been gradually identified in various diseases, including cancers [28]. It has been verified that circRNAs take part in the progression of kinds of cancer, particularly in GC [29,30]. Synchronously, PTX-based chemotherapy is very effective in the treatment of GC, but the drug resistance is a primary obstacle for the successful treatment of GC patients [4,31]. Noteworthy, circRNAs have been reported to be closely related to the drug resistance in the multiform types of tumor [32]. A prior report suggested that circ-PVT1 expression was frequently up-regulated and indicated a poor prognosis in GC [11]. However, the mechanism of circ-PVT1 in PTX resistance is still obscure in GC.

In our study, circ-PVT1 level was first examined in PTX-resistant GC tissues and cells. The data exhibited that circ-PVT1 were highly expressed in both PTX-resistant GC tissues and cells compared with their respective control groups, suggesting that circ-PVT1 might promote the PTX resistance of GC. Moreover, relevant literature confirmed that circ-PVT1 was up-regulated in doxorubicin and cisplatin-resistant osteosarcoma cells and acted as an oncogene to accelerate chemotherapy resistance of osteosarcoma cells [12]. Therefore, the effect of circ-PVT1 on PTX resistance of GC cells was further assessed. P-gp, a member of ATP-binding cassette transporters, could reduce the concentration of anti-tumor drugs in the cell, thereby leading to MDR and chemotherapy failure [33]. GST-π has been confirmed to boost drug resistance by lessening the effects of toxic substance on cells [34]. Our study proved that circ-PVT1 down-regulation resulted in a remarkable decrease in P-gp and GST-π levels. In other words, the knockdown of circ-PVT1 might mitigate the drug resistance in GC cells. Functionally, low expression of circ-PVT1 induced PXT-caused apoptosis and curbed PXT-triggered invasion in PXT-resistant GC cells *in vitro*. Interestingly, we observed that circ-PVT1 silencing suppressed GC cell growth, and knockdown of circ-PVT1 elevated PTX-caused anti-tumor effect *in vivo*, meaning that chemoresistance of circ-PVT1 was validated on GC xenografts in nude mice. That is to say, circ-PVT1 awarded PXT resistance in PXT-resistant GC cells *in vitro* and *in vivo.* Consistent with our results, circ-PVT1 was up-regulated in GC tissues and cells, and circ-PVT1 knockdown could repress the proliferation of GC cells [11].

In recent several years, accumulative studies have suggested that circRNAs could exert their function by interacting with miRNA [26]. In the present study, we discovered that miR-124-3p was expressed at low level in PXT-resistant GC tissues and cells. Intriguingly, circ-PVT1 was first confirmed to interact with miR-124-3p and inversely regulate its expression. Notably, miR-124-3p deficiency partly abrogated sh-circ-PVT1-caused PTX sensitivity in PTX-resistant GC cells. The promoting effect of miR-124-3p on drug sensitivity was also confirmed in breast cancer [35] and chronic myeloid leukemia [18].

Up to date, circRNA could act as a miRNA sponge to release mRNA [36]. In this research, ZEB1 was proved to be the target of miR-124-3p and circ-PVT1 improved ZEB1 expression by regulating miR-124-3p. Furthermore, suppression of ZEB1 reduced PXT resistance in PTX-resistant GC cells, in accordance with ovarian carcinoma cells [22]. Mechanism analysis further discovered that ZEB1 knockdown improved PTX sensitivity in PTX-resistant GC, and miR-124-3p deletion partly abolished sh-ZEB1-induced PTX sensitivity in PTX-resistant GC cells. In addition, some studies also verified that high expression of ZEB1 expedited the development of drug resistance in prostate cancer [37] and non-small-cell lung cancer [38].

In summary, our research findings disclosed that circ-PVT1 served as a sponge of miR-124-3p to up-regulate ZEB1 expression, thereby promoting PTX resistance of GC cells. Targeting circ-PVT1 provides a promising therapeutic target for GC treatment.

## Abbreviations

DMEM: Dulbecco’s modified Eagle’s medium
GC: gastric cancer
GES-1: gastric epithelial cell line
GST-π: glutathione S-transferase
P-gp: P-glycoprotein
PTX: Paclitaxel
RT-qPCR: quantitative real-time polymerase chain reaction
ZEB1: Zinc finger E-box binding homeobox 1
